# Outbreak of vancomycin-resistant *Enterococcus faecium* clone ST796, Switzerland, December 2017 to April 2018

**DOI:** 10.2807/1560-7917.ES.2018.23.29.1800351

**Published:** 2018-07-19

**Authors:** Nasstasja Wassilew, Helena MB Seth-Smith, Eveline Rolli, Yvonne Fietze, Carlo Casanova, Urs Führer, Adrian Egli, Jonas Marschall, Niccolò Buetti

**Affiliations:** 1 Department of Infectious Diseases, University Hospital Bern, Bern, Switzerland; 2 Division of Clinical Microbiology, University Hospital Basel, Basel, Switzerland; 3 Applied Microbiology Research, Department of Biomedicine, University of Basel, Basel, Switzerland; 4 Institute for Infectious Diseases, University of Bern, Bern, Switzerland; 5 Infectious Diseases Department, Biel Hospital, Biel, Switzerland

**Keywords:** VRE, vancomycin-resistant enterococci, ST796, outbreak, emergent clone, enterococcus, whole genome sequencing

## Abstract

A large outbreak of vancomycin-resistant enterococci (VRE) is affecting four hospitals in the Canton of Bern, Switzerland, since December 2017. Of 89 cases identified as carriers, 77 (86.5%) VRE isolates were virtually indistinguishable using whole genome sequencing, and identified as multilocus sequence type (MLST) ST796. This clone, previously only described in Australia and New Zealand, is characterised by rapid spread and the ability to cause bloodstream infections. It requires a multifaceted infection prevention effort.

Vancomycin-resistant enterococci (VRE) are multidrug-resistant microorganisms that cause healthcare-associated infections and are associated with an increased risk of mortality and length of hospital stay [1,2]. An outbreak with VRE affected several hospitals in the Canton of Bern since December 2017, with a new VRE clone ST796 characterised by a rapid intra- and inter-hospital dissemination. This clone has recently emerged in Australia and New Zealand and has quickly spread between hospitals [3]; VRE ST796 has not yet been described in Europe. Here, we describe a large outbreak of this clone across multiple hospitals in Switzerland and illustrate the genetic relationship of the Swiss and Australian isolates.

## Description of the outbreak and outbreak strain analysis

The data reported here were obtained from 30 December 2017 to 30 April 2018. An outbreak investigation was started when two cases of VRE *E. faecium* bloodstream infection were reported on the haemato-oncology ward of Bern University Hospital on 30 December 2017.

Colonies from selective media identified as *E. faecium* with Matrix Assisted Laser Desorption/Ionisation Time-of-Flight (MALDI-TOF) mass spectrometry (Bruker Daltonics, Bremen, Germany) were screened (Xpert vanA/vanB, Cepheid, Sunnyvale, California (CA), United States (US)) for rapid detection of *vanA* and *vanB* resistance genes before phenotypic susceptibility testing by Clinical Laboratory Standards Institute (CLSI) disk diffusion and Etest (bioMérieux, Marcy l’Étoile, France). All VRE isolates were analysed by whole genome sequencing (WGS) to support epidemiological linkage (Supplement 1). WGS sequencing was performed using a MiSeq Illumina platform (accredited with ISO 17025 norm at the Division of Clinical Microbiology, University Hospital Basel) with 2 x 300nt paired-end sequencing after Nextera XT library preparation. The resulting reads were de novo assembled and analysed by core genome multilocus sequence typing (cgMLST) within Ridom SeqSphere Software (version 4.1.6). Additional ST796 strain genome sequences were downloaded from databases for comparison [4,5]. All read data have been deposited with the European Nucleotide Archive (ENA) (https://www.ebi.ac.uk/ena) under the project number PRJEB27159.

**Figure 1:**
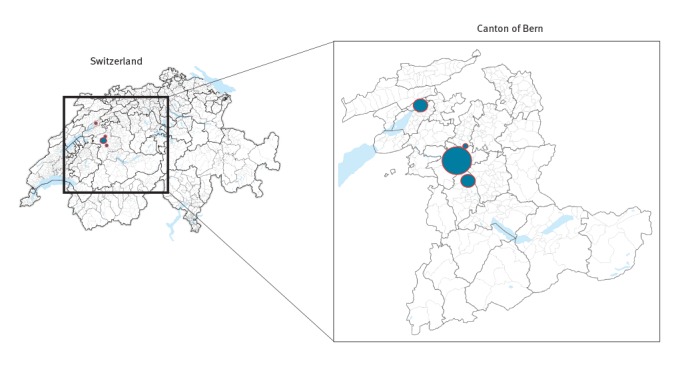
Distribution of vancomycin-resistant *Enterococcus faecium* ST796 in four different hospitals, Canton of Bern, Switzerland, 30 December 2017 to 30 April 2018 (n = 89)

Three of six hospitals within the Bern University Hospital group were affected (one university hospital, one community hospital, one rehabilitation centre), as well as a community hospital about 40 km away from Bern, in the university hospital’s catchment area (Figure 1).

The largest outbreak at University hospital is shown by the largest circle.

**Figure 2:**
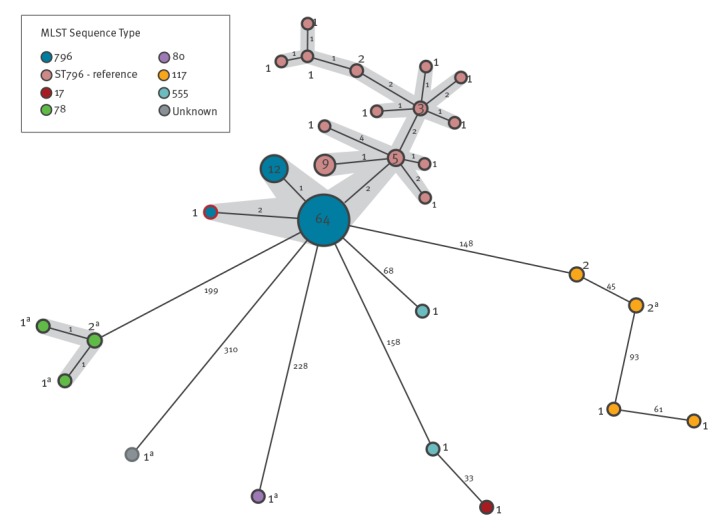
Core genome multilocus sequencing typing multiple spanning tree of *Enterococcus faecium* isolates, the Canton of Bern outbreak, Switzerland and isolates detected in Australia, 30 December 2017–30 April 2018

As at April 2018, 3,096 screening samples were obtained from contact patients and 89 patients were found to be colonised or infected with VRE (Table 1). In these 89 patients, 77 (86.5%) of isolates were found to be virtually indistinguishable by cgMLST (separated by up to three alleles) and identified as MLST type ST796 (Figure 2). All of these carried the resistance type *vanB*.

**Table 1:** Description of vancomycin-resistant *Enterococcus faecium* ST796 outbreak isolates in four different hospitals, Canton of Bern, Switzerland, 30 December 2017–30 April 2018, (n= 89)

	All patients (N = 89)		VRE isolates ST796 (n = 77)		Other VRE isolates ST^a,b^ (n = 15)	
	n	%	n	%	n	%
Mean age (median)	67.9	69	68.9	71	62.1	64.5
Female	38	42.7	34	44.2	3	20
Resistance type vanB	81	91	77	100	7	46.7
University hospital	74	83.1	63	81.8	14	93.3
Haemato-oncology	28	31.5	27	35.1	2	13.3
Invasive infection^c^	7	7.9	7	9.1	1	6.7
BSI	5	5.6	5	6.5	0	0
Community hospital 1	2	2.2	2	2.6	0	0
Community hospital 2	9	10.1	8	10.4	1	6.7
Rehabilitation centre	4	4.5	4	5.2	0	0

BSI: bloodstream infection; ST: sequence type; VRE: vancomycin-resistant enterococci.

^a^ The VRE isolate was not sequenced in one patient. Four patients had dual infection.

^b^ MLST types ST117, ST555, ST78, ST17, ST80.

^c^ Bloodstream infections (n = 5); abdominal abscess (n = 1); deep wound infection (n = 1). All invasive isolates were detected in the university hospital.

The core genome multilocus sequencing typing (cgMLST) analysis is based on 1423 loci [14] and was performed in Ridom Seqsphere v4.1.6.

Number of isolates belonging to each cluster is shown in the circles and the number of allele differences between clusters is presented on the connecting lines. Outbreak strains are shown in dark blue*, with the clonal complex highlighted in grey. Reference ST796 strains from Australia are shown in pink.

^a^ Isolates carrying vanA.

In six patients, VRE was initially detected in clinical samples (and not in screening samples), of whom five carried the outbreak strain ST796. Of 77 patients colonised or infected with VRE ST796, the mean age was 68.9 years, 34 (44%) were female, seven (7.9%) developed an invasive infection: five (6.5%) bacteraemia, one (1.3%) abdominal abscess and one (1.3%) deep wound infection. Temporo-spatial links were found for almost all patients in whom the outbreak strain was detected during the course of the outbreak (i.e. patients had shared a room or were treated by the same healthcare worker).

Of the 77 ST796 isolates, antimicrobial susceptibility profiles were obtained from 68 and showed resistance to ampicillin, levofloxacin and high-level resistance to gentamycin, but not streptomycin. Forty-six isolates were resistant to vancomycin (minimum inhibitory concentration (MIC) > 16mg/L), 21 were intermediate (MIC 8–16mg/L) and one tested susceptible (4mg/L) (median MIC 32 mg/L). All isolates were susceptible to teicoplanin (median MIC 0.5mg/L). The remaining isolates were identified as MLST type ST117 (n = 6), ST555 (n = 2), ST78 (n = 4), ST17 (n = 1) and ST80 (n = 1). Only eight isolates detected during this period carried *vanA* (Figure 2).

The genomes of the Swiss outbreak isolates (ST796) all map to > 97% of the reference genome Ef_aus0233 from Australia, separated by ca 27 single nucleotide polymorphisms (SNPs), some of which cluster, indicative of recombination (Supplement 2 and Figure 2).

## The outbreak management strategy

After the outbreak was declared on 4 January 2018, a VRE outbreak team was formed. A large outbreak investigation and management strategy was started immediately, including temporary admission stops in affected wards. Patients colonised or infected with VRE were cohorted and placed under contact precautions; staff cohorting was also implemented. Contact patients of VRE positive patients were retrospectively identified, screened on a weekly basis (3 negative screenings (0, 7, 14 days if hospitalised) and pre-emptively placed under contact precautions or if already discharged tagged in an electronic alarm system. Environmental disinfectant cleaning was implemented and intensified where VRE transmissions had been identified. Wards that had housed patients colonised with VRE were subject to cross-sectional screening. Hand hygiene compliance was reinforced on every occasion.

**Figure 3:**
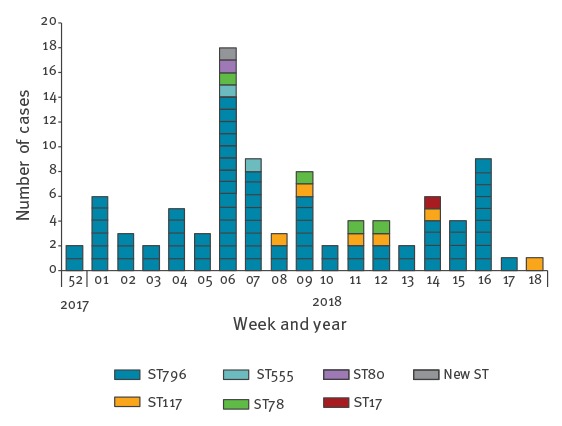
Epidemic curve of vancomycin-resistant enterococci (VRE) cases by sequence type, Canton of Bern outbreak, Switzerland, December 2017–April 2018 (n = 89)

With these measures in place, an initial decrease in new detections of VRE cases during the follow-up period was observed (January 2018–April 2018). The epidemiologic curve of VRE infected or colonised patients is shown in Figure 3.

## Discussion

The World Health Organization (WHO) has listed VRE as a pathogen with high priority in its global priority list of antibiotic-resistant bacteria [6]. While the incidence of VRE varies between countries, the European mean vancomycin resistance prevalence was 11.8% in *E. faecium*, in 2016 [7]. Transmission takes place via the environment (i.e., surfaces or medical devices) or healthcare workers and numerous outbreaks have been documented worldwide [8,9].

To the best of our knowledge, we report the first description of VRE ST796 clonal dissemination across several hospitals in Europe. *E. faecium* ST796 was first recognised at an Australian hospital during 2011 [10] and spread rapidly throughout Australia and New Zealand hereafter. In 2013, it was identified as the cause of a large clonal outbreak of VRE in a neonatal unit of a hospital in Victoria [11]. In the following years, it spread from two Melbourne hospitals to hospitals in South Australia, New South Wales and New Zealand. VRE ST796 was shown to have largely replaced the previously predominant clone ST203 in a Melbourne hospital, by 2014 [3]. The following year, ST796 was found to be responsible for 53% of all *E. faecium* VRE bacteraemias in Melbourne hospitals [5]. In 2015, a marked increase in VRE notifications occurred In Tasmania and, in 2016, ST796 had become the most important clone isolated at the major tertiary referral centre of this island [4]. Moreover, in 2016, ST796 was one of the most frequently detected VRE isolates among bacteraemic episodes in Australia [12]. ST796 *E. faecium* was described to cause colonisation with low rates of clinical infection [11], however, a number of invasive VRE infections were also described in other hospitals [3].

The rapid inter-hospital spread across states and countries implies an effective transmission ability of this clone [3] and it does not appear that emerging healthcare-associated strains of *E. faecium* depend on enhanced antibiotic resistance for their success [3]. The complete genome of a representative *E. faecium* isolate ST796 (Ef_Aus0233) was examined and used to describe the evolution from an ST555 to ST796 *E. faecium* isolate by means of several genomic events. This indicates the high propensity of the hospital *E. faecium* lineage to change, presumably in response to specific hospital environments [5]. An Australian group investigating hospital *E. faecium* isolates found increased tolerance to alcohol in isolates provided from 2010 onwards as compared with older isolates. They observed a positive association between time and increasing alcohol use, suggesting a high propensity of the bacteria to adapt under selective pressure – in this case possibly triggered by the use of alcohol based wipes [13].

The WGS analyses revealed a clear genomic relationship between isolates from the outbreak in the Canton of Bern described here and those from Australia. The rapid spread of this multidrug-resistant clone may seriously endanger healthcare facilities and warrants strengthening and synchronisation of national infection control practices. In Switzerland, VRE incidence is not systematically monitored and national surveillance data are lacking. In collaboration with the National Center of Infection Control (Swissnoso), and the Swiss Centre for Antibiotic Resistance (ANRESIS) a national programme on VRE epidemiology and the corresponding outbreak management has been launched in Switzerland on 29 June 2018.

## *Authors’ correction

In Figure 2, in the figure note, outbreak strains were mistakenly labelled as ‘red’. This was changed to ‘dark blue’ on 20 July 2018 as requested by the authors.
