# Authors’ correction for Euro Surveill. 2018;23(29)

**DOI:** 10.2807/1560-7917.ES.2018.23.30.1807263

**Published:** 2018-07-26

**Authors:** Eurosurveillance editorial team

**Affiliations:** 1 European Centre for Disease Prevention and Control (ECDC), Stockholm, Sweden

As requested by the authors of the article ‘Outbreak of vancomycin-resistant *Enterococcusfaecium* clone ST796, Switzerland, December 2017 to April 2018’, published on 19 July 2018, the label ‘red’ for outbreak strains was corrected to ‘dark blue’ in the note for Figure 2. This mistake was corrected on 20 July 2018.

